# Distinct Age-Dependent C Fiber-Driven Oscillatory Activity in the Rat Somatosensory Cortex

**DOI:** 10.1523/ENEURO.0036-20.2020

**Published:** 2020-10-07

**Authors:** Pishan Chang, Lorenzo Fabrizi, Maria Fitzgerald

**Affiliations:** Department of Neuroscience, Physiology and Pharmacology, University College London, London WC1E6BT, United Kingdom

**Keywords:** brain development, brain oscillations, C fibers, EEG, pain, somatosensory cortex

## Abstract

When skin afferents are activated, the sensory signals are transmitted to the spinal cord and eventually reach the primary somatosensory cortex (S1), initiating the encoding of the sensory percept in the brain. While subsets of primary afferents mediate specific somatosensory information from an early age, the subcortical pathways that transmit this information undergo striking changes over the first weeks of life, reflected in the gradual emergence of specific sensory behaviors. We therefore hypothesized that this period is associated with differential changes in the encoding of incoming afferent volleys in S1. To test this, we compared S1 responses to A fiber skin afferent stimulation and A + C skin afferent fiber stimulation in lightly anaesthetized male rats at postnatal day (P)7, P14, P21, and P30. Differences in S1 activity following A and A + C fiber stimulation changed dramatically over this period. At P30, A + C fiber stimulation evoked significantly larger γ, β, and α energy increases compared with A fiber stimulation alone. At younger ages, the changes in S1 oscillatory activity evoked by the two afferent volleys were not significantly different. Silencing TRPV1+ C fibers with QX-314 significantly reduced the γ and β S1 oscillatory energy increases evoked by A + C fibers, at P30 and P21, but not at younger ages. Thus, C fibers differentially modulate S1 oscillatory activity only from the third postnatal week, well after the functional maturation of the somatosensory cortex. This age-related change in afferent evoked S1 oscillatory activity may underpin the maturation of sensory discrimination in the developing brain.

## Significance Statement

Behavioral responses to sensory stimulation of the skin undergo major developmental changes over the first postnatal weeks. Here, we show that this is accompanied by a shift in the differential frequency encoding of sensory A fiber and C fiber afferent inputs into the developing rat somatosensory cortex. The results demonstrate major postnatal changes in the ability of the cortex to differentiate between afferent sensory inputs arriving in the mammalian brain.

## Introduction

Distinct temporal rhythms of neural activity are an essential part of communication in the brain. In both humans and rodents, tactile and nociceptive sensory stimulation is associated with alterations in these patterns (Pfurtscheller and Lopes da Silva, 1999; Stančák, 2006; Peng and Tang, 2016). The primary somatosensory cortex (S1) plays a key role in perceptual recognition of the presence, location, intensity, submodality and quality of touch, thermal sensibility, and pain (Vierck et al., 2013) and the oscillatory rhythms commonly recorded over the rat somatosensory cortex (S1) are selectively modified by sensory stimulation. For example, the amplitudes of both α and β rhythms in rat S1 are suppressed by a tactile stimulus, with a comparable time course to humans (Fransen et al., 2016), and noxious skin stimulation results in selective increases in S1 γ activity in both rodents and human subjects (Hu and Iannetti, 2019). γ Activity reflects stimulus encoding in S1 (Yue et al., 2020) and experimental induction of S1 γ oscillations via optogenetic activation of parvalbumin-expressing inhibitory interneurons enhances nociceptive sensitivity and induces aversive avoidance behavior (Tan et al., 2019). Changes in other oscillatory frequencies are also reported following skin stimulation, including decreases in α and β energy related to cutaneous stimulus intensity (Nickel et al., 2017) and synchronization of θ-α energy following noxious stimulation (Peng et al., 2018). The ability of particular modalities of afferent input to differentially modulate the temporal rhythms in S1 may be an important mechanism underpinning the communication of sensory discrimination in the brain.

Behavioral reflex recording in newborn rodents and humans reveal that somatosensory discrimination is poorly developed such that “nociceptive” reflexes are indistinguishable from those evoked by innocuous touch (Fitzgerald et al., 1988; Cornelissen et al., 2013; Green et al., 2019). The underlying modality-specific connections in the spinal cord also develop postnatally, characterized by structural and functional refinement of A fiber terminals and their synaptic inputs (Coggeshall et al., 1996; Fitzgerald and Jennings, 1999; Beggs et al., 2002; Granmo et al., 2008), gradual strengthening of C fiber synaptic connections (Baccei et al., 2003; Fitzgerald, 2005; Walker et al., 2007), and maturation of dorsal horn inhibitory circuitry (Baccei and Fitzgerald, 2004; Koch et al., 2012), accompanied by the onset of selective descending brainstem control of A and C fiber evoked (Koch and Fitzgerald, 2014) and tactile and noxious evoked (Schwaller et al., 2017) dorsal horn activity, all of which accompany the segregation of innocuous and noxious cutaneous reflexes over the postnatal period (Baccei and Fitzgerald, 2013).

The delayed postnatal emergence of modality selective dorsal horn circuitry is likely to impact on the sensory modulation of oscillatory rhythms in S1. Indeed there is evidence in human infants of a transition in brain response following tactile and noxious stimulation from nonspecific, evenly dispersed neuronal bursts to modality-specific, localized, evoked potentials (EPs), suggesting an emergence of specific neural circuits necessary for discrimination between touch and nociception (Fabrizi et al., 2011). Temporal patterns of cortical communication develop gradually in rodents over the first postnatal weeks (Khazipov et al., 2004; Minlebaev et al., 2011; Mitrukhina et al., 2015; Chang et al., 2016), but the maturation of selective modification of these rhythms by different afferent inputs is not known.

We hypothesized that the first postnatal weeks are associated with changes in the encoding of incoming afferent volleys in S1 and specifically that myelinated A fiber and unmyelinated C fiber cutaneous afferents only differentially modulate S1 oscillatory activity after two postnatal weeks. To test this, we compared the effect of A fiber and C fiber skin afferent fiber stimulation on S1 oscillatory activity in lightly anaesthetized male rats at postnatal day (P)7, P14, P21, and P30. S1-EPs and oscillatory rhythms were recorded at each age, and the ability of C fiber versus A fiber cutaneous afferent input to differentially modulate the temporal rhythms in S1 was evaluated by activation or selective silencing techniques.

## Materials and Methods

### Animals and ethics

All experiments were performed in accordance with the United Kingdom Animal (Scientific Procedures) Act 1986. Reporting is based on the ARRIVE Guidelines for Reporting Animal Research developed by the National Centre for Replacement, Refinement and Reduction of Animals in Research, London, United Kingdom. Male Sprague Dawley rats aged P7, P14, P21, and P30, were obtained from the Biological Services Unit, University College London. Rats were housed in controlled conditions in accordance with guidance issued by the Medical Research Council in Responsibility in the Use of Animals for Medical Research (1993), and all experiments were conducted under License from the United Kingdom Home Office and with Local Ethical Review panel approval. All animals were from the same colony, bred and maintained in-house, and exposed to the same caging, diet, and handling throughout development. Rats were housed in cages of four age-matched animals (P30) or with the dam and littermates (P7, P14, and P21) under controlled environmental conditions (24–25°C; 50–60% humidity; 12/12 h light/dark cycle) with free access to food and water. Animals of both sexes were randomly picked from litters for recording. Treatment groups were distributed across multiple litters and/or adult cage groups.

### Recording from the S1

Electrophysiological recordings were conducted under general anesthesia. Rats were initially anaesthetized with 4% isoflurane (Abbot, AbbVie Ltd.) in 100% O_2_ (flow rate of 1–1.5 l/min) and trachea cannulated (P7: 27 gauge; P14: 18 gauge; P21: 18 gauge; P30: 16 gauge). They were then placed in a stereotaxic frame and the cannula connected immediately to a mechanical ventilator (Bioscience) and calibrated isoflurane vaporizer (Harvard Apparatus). Anesthesia was adjusted to 2–3% isoflurane in 100% O_2_ (1–1.5 l/min) and a craniotomy performed to expose the surface of the cerebral cortex. Body temperature was maintained with a thermostatically controlled heated blanket, and the electrocardiogram was monitored throughout (Neurolog, Digitimer). A recording electrode (stainless-steel 25 mm, E363/1, Plastics One Inc.) was inserted into the S1 in the somatotopic region for the hindpaw. Coordinates for P7 and P14 rats were lateral 2.0 mm from midline and posterior 0.5 mm from the bregma; and for P21 and P30 rats were lateral 2.5 mm from midline, and posterior 1 mm from the bregma (Kalliomäki et al., 1993; Paxinos and Watson, 2013; Paxinos et al., 2013). The reference electrode was placed subcutaneously on the surface of the skull anterior to the bregma, and the ground electrode was placed subcutaneously in the back. The depth of the recording electrode was adjusted to optimize the EP amplitude and the recording depth verified histologically to be in layer 5–6 of the somatosensory cortex as described previously (Chang et al., 2016). The isoflurane concentration was then reduced to 1% and allowed to equilibrate for at least 30 min before recording began. Continuous activity was recorded under 1% isoflurane using a Neurolog NL100 headstage connected to a NL104 amplifier and a low pass NL125 filter (100 Hz). The signal was sampled at 16K Hz using Molecular Devices (Digidata 1400A, Molecular Devices). Data were acquired and stored using a Windows PC based program, WinEDR v3.3.6 (John Dempster, University of Strathclyde, United Kingdom) for later analysis.

The recording parameters were confirmed in P30 animals by recording LFP following plantar hindpaw electrical stimulation under 1% isoflurane. Clear EPs were recorded following contralateral, but not ipsilateral hindpaw stimulation, consistent with previous studies (Welker, 1971; Killackey, 1973). Increasing current intensity [500-μs square pulses of 3.2 μA, 32 μA, 320 μA, 3.2 mA; 10 stimuli with a 10-s interstimulus interval (ISI) in *n* = 6 animals] established the threshold required to elicit optimal EPs in S1 to be 3.2 mA at P30.

### Hindpaw skin stimulation

Electrical stimulation was applied through two stainless steel pin electrodes placed subcutaneously 3–5 mm apart in the plantar skin of the contralateral hindpaw, using a constant current stimulator (Neurolog, Digitimer). Different pulse widths were used to recruit the following afferent fiber groups: Aβ and Aδ fibers (50 μs, 3.2 mA) or Aβ, Aδ, and C-fibers (500 μs, 3.2 mA; Jennings and Fitzgerald, 1998; Koch and Fitzgerald, 2014). Stimuli were applied with a 10-s ISI, 10 times at A fiber intensity and 10 times at A + C fiber intensity, separated by an interval of 5 min. The afferent groups excited by these peripheral electrical stimulation intensities have been previously established from recording nerve compound action potentials (CAPs; Koch and Fitzgerald, 2014) and for simplicity and clarity are denoted “A fiber” and “A + C fiber” stimulation intensities throughout.

### C-fiber silencing

C-fibers were silenced using a combination of QX-314 and capsaicin, which effectively blocks action potential generation by insertion of the membrane impermeable sodium channel blocker QX-314 into C fiber afferents via the TRPV1 channel pore (Binshtok et al., 2007; Puopolo et al., 2013). The combination of QX-314 (lidocaine N-ethyl bromide; 2%, Tocris Bioscience) and capsaicin (0.1%, Sigma) with solvent (20% ethanol, 7% Tween 80, and 70% normal saline) was prepared and stored at −20°C and injected into the plantar hindpaw (P14: 5 μl; P21 and P30: 10 μl).

The effectiveness of C fiber block on heat and mechanical nociception was tested at P14, P21, and P30. For heat nociception, we used the Hargreaves test in awake animals, while for mechanical nociception, we measured the electromyogram (EMG) of the withdrawal reflex to plantar pinch in lightly anaesthetized animals (1% isoflurane). QX-314/capsaicin caused an increase in withdrawal latency to heat stimulation compared with baseline, peaking at 60–150 min after injection and lasting over 3 h. Friedman test with Dunn’s multiple comparisons test (*n* = 8 at all ages): χ^2^(6) at P14 = 19.02 (*p* = 0.004); χ^2^(6) at P21 = 26.46 (*p* < 0.001); χ^2^(6) at P30 = 25.82 (*p* < 0.001). QX-314/capsaicin also caused a significant decrease in EMG amplitude to pinch peaking between 60–150 min [Wilcoxon test (*n* = 8 at all ages): W at P30 = −36 (*p* = 0.008); W at P21 = −45 (*p* = 0.004); W at P14 = −36 (*p* = 0.008)].

### Experimental design and statistical analysis

#### Time domain analysis

Peak EP amplitude and onset latency for each animal/condition were measured following electrical stimulation. Continuous recordings were segmented into epochs of 1.5 s, from 0.15 s prestimulus to 1.35 s poststimulus. Epochs were then baseline corrected and averaged for each animal (10 stimuli per animal) and peak EP amplitudes identified.

#### Frequency domain analysis

Energy changes in LFP in different frequency bands were calculated using the Hilbert transform in the Brainstorm MATLAB toolbox (Le Van Quyen et al., 2001; Bruns, 2004; Tadel et al., 2011). This filters the signals in various frequency bands with a bandpass filter and then computes the Hilbert transform of the filtered signal. The magnitude of the Hilbert transform of a narrow-band signal is a measure of the envelope of this signal, and therefore gives an indication of the activity in this frequency band. Energy was then calculated by squaring the magnitude of the Hilbert transform. Continuous recordings were segmented into 10 s epochs, from 5 s prestimulus to 5 s poststimulus. Each epoch was then filtered in various frequency bands with bandpass filters for δ (2–4 Hz), θ (5–7 Hz), α (8–12 Hz), β (15–29 Hz), and γ (30–90 Hz) band. The Hilbert transform of each filtered signal was then computed and averaged within time bins of 0.5 s (0–500, 500–1000, and 1000–1500 ms poststimulus). These were then averaged across repetitions within each animal. Stimulus-induced energy changes for each animal were then calculated by normalizing the poststimulus values by a baseline time bin which did not include a spontaneous activity burst (which may have occurred just before the stimulus). Stimulus-induced energy changes specifically associated with C fiber recruitment were estimated by comparing energy changes following A + C versus A fiber stimulation (A + C/A ratio). We then estimated the stimulus-induced energy changes following A + C stimulation before and 60–150 min after administration of QX-314/capsaicin and compared them (A + C after-/before-QX-314 ratio) to identify changes in activity patterns caused by the silencing of C fibers.

#### Statistical analysis

Statistical analysis of EP peak amplitude, onset latency, and energy changes was performed using GraphPad Prism 6 (GraphPad Software) and MATLAB. All data are presented as mean ± SEM. Mean peak EP amplitude and onset latency were compared across ages and stimulus intensities (condition) with a two-way mixed ANOVA [within-subjects: stimulus intensity (condition); between-subjects: age] followed by Tukey’s multiple comparisons test.

Paired differences in energy from (1) A + C fiber versus A fiber stimulation and (2) after versus before QX-314 treatment were computed for each frequency band and poststimulus time bin, at each age. These differences were statistically analyzed using estimation statistics, reporting mean differences (effect size) with expressions of uncertainty (confidence interval estimates). To do this, we directly introduced the raw data into an open source estimation program available on https://www.estimationstats.com (Ho et al., 2019) and downloaded the results and graphs. In this method, each paired mean difference is plotted as a bootstrap sampling distribution, using 5000 bootstrap samples and the confidence intervals are bias corrected and accelerated. The *p* value(s) reported are the likelihood(s) of observing the effect size(s), if the null hypothesis of zero difference is true. For each permutation *p* value, 5000 reshuffles of the control and test labels were performed; *p* < 0.05 is considered a significant difference.

## Results

### Recruiting C fibers does not change S1 local field EP amplitudes at any age

**Figure 1.**
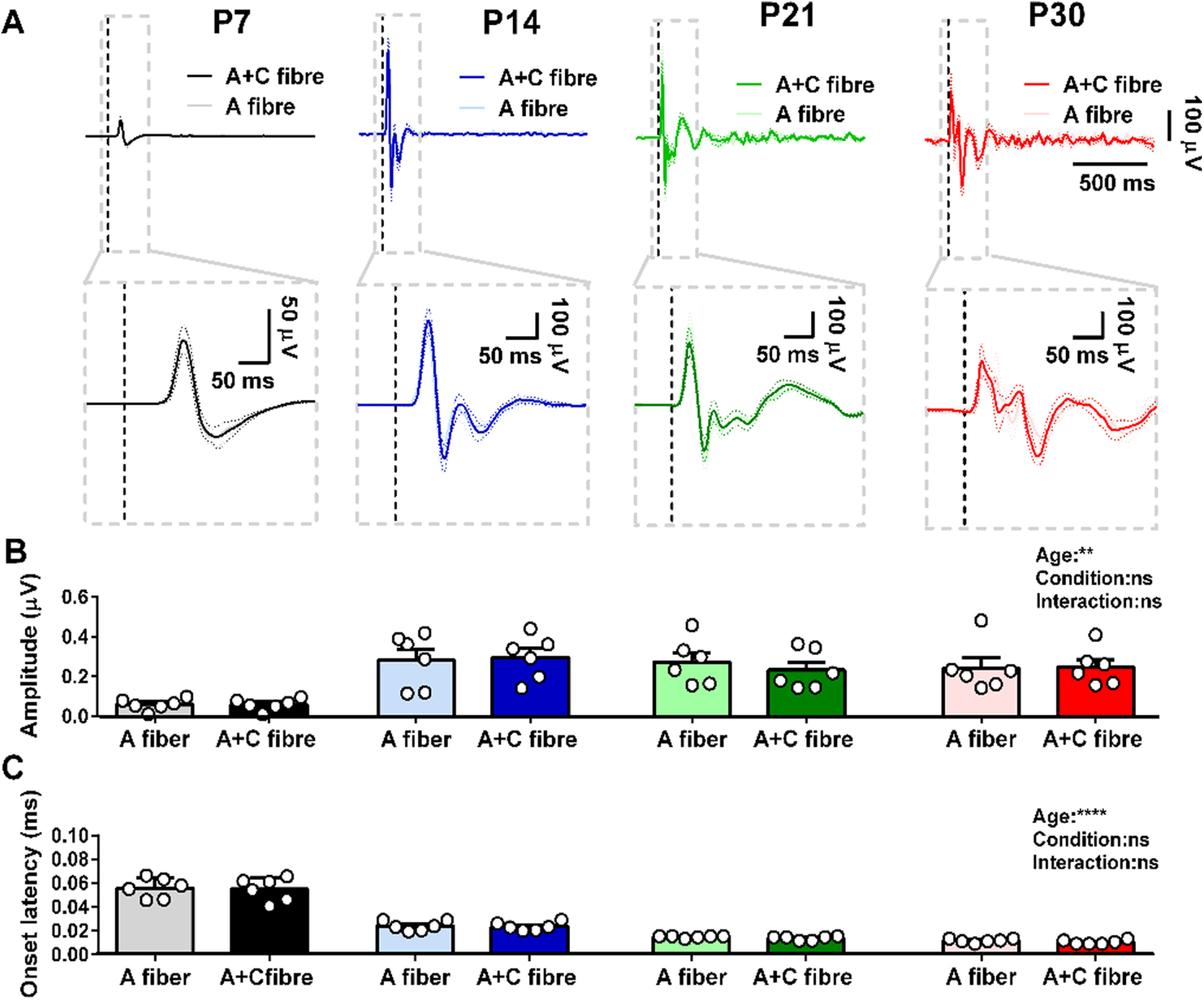
Maturation of evoked local field potentials (EPs) in S1 following contralateral hindpaw stimulation. ***A***, Average EPs following A fiber (50 μs) and A + C fiber (500 μs) electrical stimulation of the contralateral hindpaw at postnatal day (P)7, P14, P21, and P30 (mean ± SEM, 10 stimuli at 10-s ISI, 3.2 mA, *n* = 6 rats at each age). Lower panels are a zoom-in view of the gray boxes in the top panels. Vertical dotted lines indicate the time of stimulation. EP peak amplitude (***B***) and onset latency (***C***) in response to electrical skin stimulation; two-way mixed ANOVA; **p < 0.01, ****p < 0.0001, and ns: not significant. Data are shown as means ± SEM. Each dot represents average of stimuli from one animal.

Local field EPs were elicited in S1 at P7, P14, P21, and P30 (young adult) in response to electrical stimulation of the contralateral hindpaw. Stimuli were applied at strengths that activate only A fibers or both A + C fibers [10 stimuli at 10-s ISI, 3.2 mA, 50 μs (A) or 500 μs (A + C), *n* = 6 at each age; Fig. 1*A*]. There was no significant difference between A fiber and A + C fiber EP peak amplitudes or onset latencies within each age (Fig. 1*B*,*C*).

### Recruiting C fibers adds a distinct, age-related component to S1 oscillatory activity

Even if local field EPs following A and A + C do not differ in the time domain, there still may be differences in the frequency content of the evoked activity which are not phase-locked to the stimulus and are therefore lost in the time average. To address this point, we compared the energy changes (relative to baseline) in the 1500-ms epoch following A fiber and A + C fiber hindpaw stimulation.

**Figure 2.**
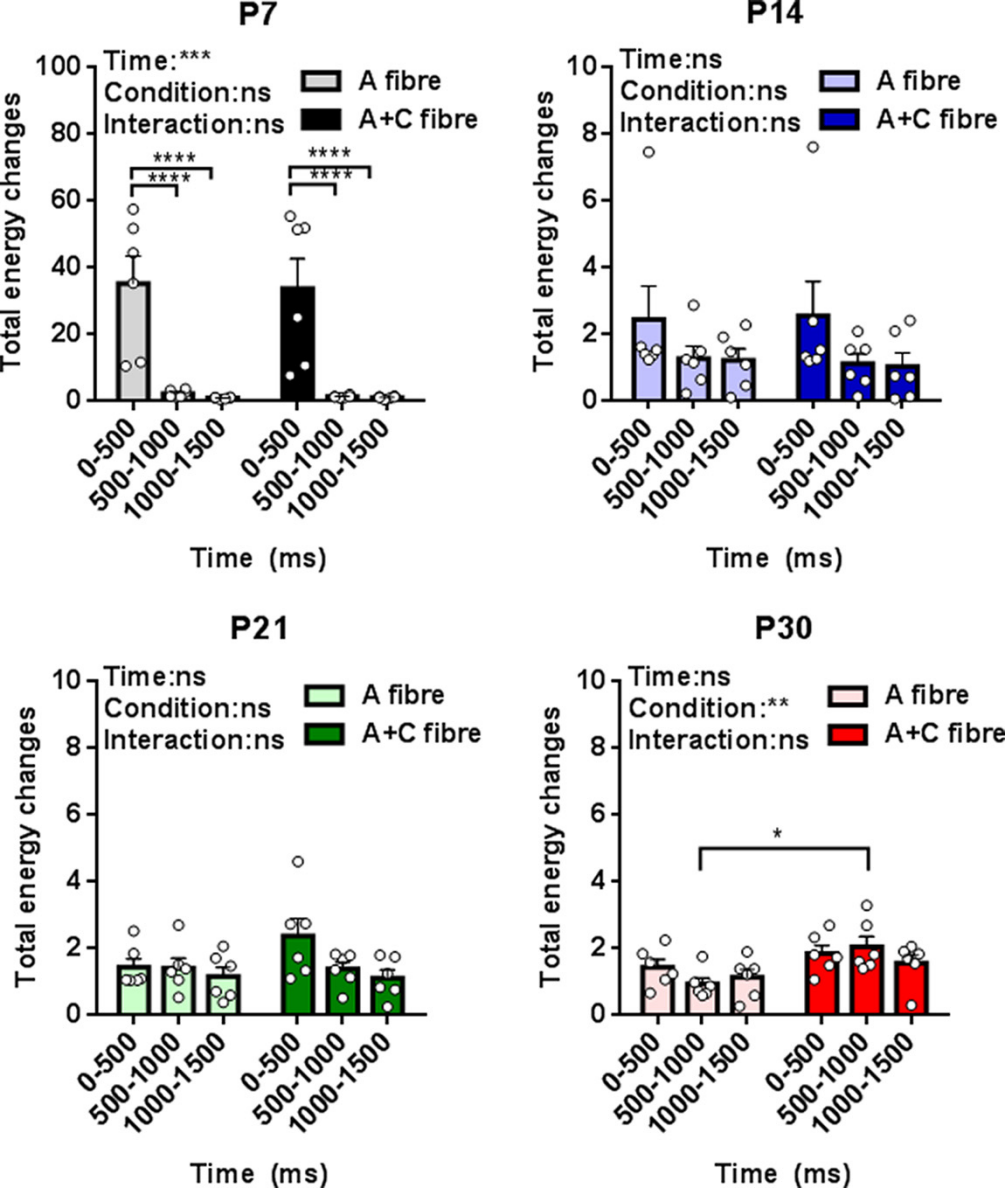
Maturation of total energy changes in neuronal oscillations in S1 following contralateral hindpaw skin stimulation. Total energy changes evoked by A fiber (50 μs) and A + C fiber (500 μs) electrical stimulation of the contralateral hindpaw at postnatal day (P)7, P14, P21, and P30 between 0 and 500, 500 and 1000, and 1000 and 1500 ms poststimulation. Mean (±SEM) total energy changes (compared with baseline) following electrical stimuli (10 stimuli at 10-s ISI, 3.2 mA, *n* = 6 rats at each age). Each dot represents the average across stimuli for each rat. Two-way RM ANOVA (summary results are shown on top left of each panel) with Sidak’s multiple comparisons test; *p < 0.05, **p < 0.01, ***p < 0.001, ****p < 0.0001, and ns: not significant.

Figure 2 shows the total energy change following hindpaw stimulation, relative to baseline, at each age. At P7 there is a large increase in energy which is restricted to the first 500 ms poststimulus. From P14 onwards, the energy changes, relative to baseline, are smaller but last for 1500 ms. When total S1 oscillatory energy evoked by A fiber and by A + C fiber stimulation were directly compared, it was clear that A + C fiber evoked total energy is only significantly greater than A fiber at P30 in the 500- to 1000-ms poststimulus time epoch, with no significant differences in total energy attributable to C fiber recruitment before that age (Fig. 2).

We next tested which frequency bands were contributing to the total energy differences between A and A + C fibers stimulation in Figure 2. To do this, we compared A and A + C energy changes within the same animal, in each frequency band: δ (2–4 Hz), θ (5–7 Hz), α (8–12 Hz), β (15–29 Hz), and γ (30–90 Hz). Data were expressed as the paired difference between A + C fiber evoked energy and A fiber evoked energy at 0–500, 500–1000, and 1000–1500 ms poststimulus, at each age. The data were analyzed using estimation statistics with a permutation *t* test (Ho et al., 2019).

**Figure 3.**
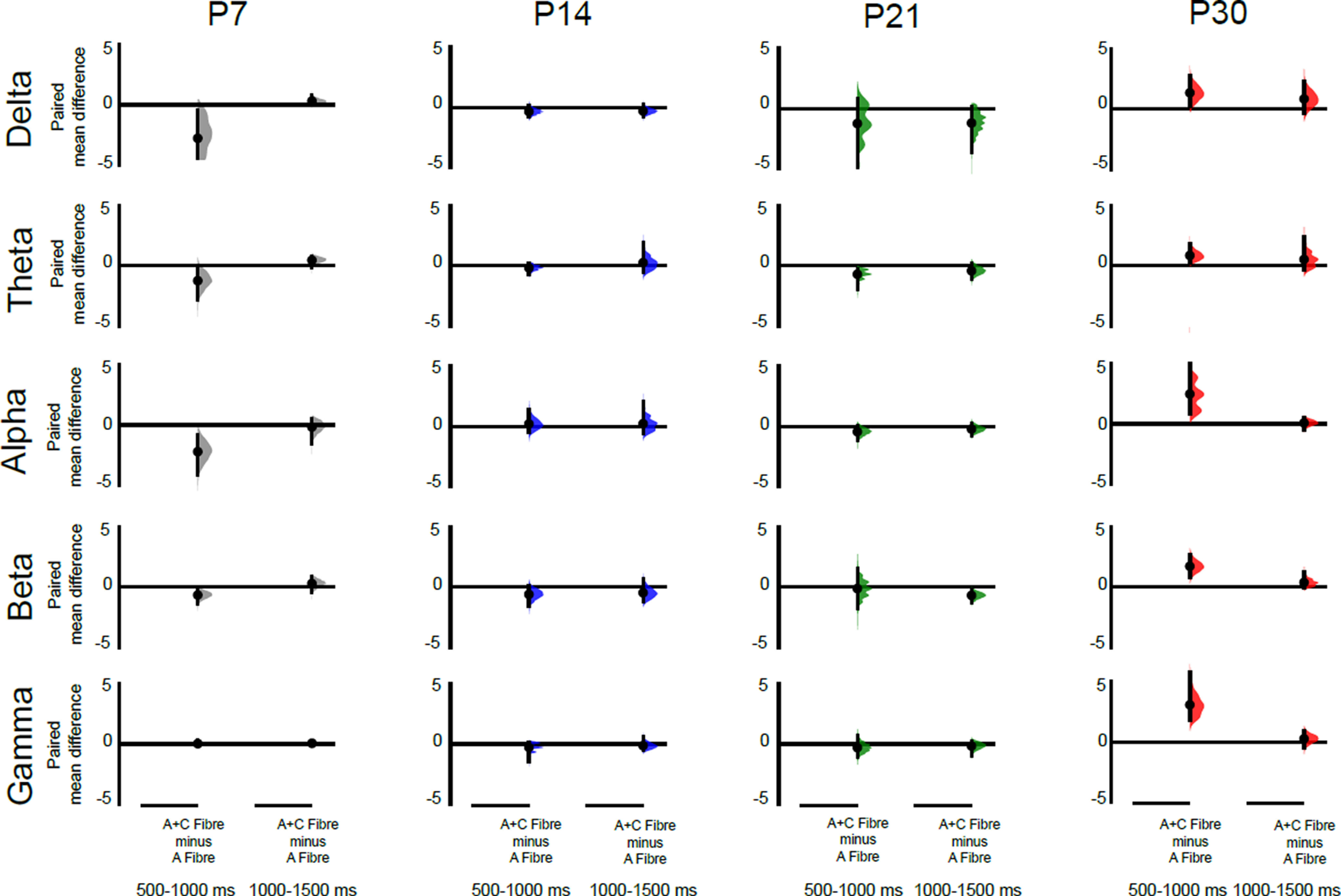
Recruitment of cutaneous C fibers evokes distinct age-related increases in S1 γ, β, and α oscillatory energy. Differences in the energy evoked by A + C fiber versus A fiber cutaneous stimulation in S1 at P7, P14, P21, and P30 (*n* = 6 animals per age). The paired mean difference at 500–1000 and 1000–1500 ms poststimulus are shown for each frequency band [δ (2–4 Hz), θ (5–7 Hz), α (8–12 Hz), β (15–29 Hz), and γ (30–90 Hz)]. Mean differences are depicted as dots; 95% confidence intervals are indicated by the ends of the vertical error bars. A positive value means indicates a greater energy following A + C fiber stimulation, compared with A fiber stimulation and can be attributed to recruitment of C fibers. Significant increases in γ, α, and β energy occur at P30, and a significant decrease in β energy occurs at P21. A shorter latency (0–500 ms) decrease in α energy at P7 is not shown. See text for effect sizes, confidence intervals, and permutation *p* values. Each paired mean difference is plotted as a bootstrap sampling distribution, using 5000 bootstrap samples, and the confidence intervals were bias corrected and accelerated (Ho et al., 2019).

**Figure 4.**
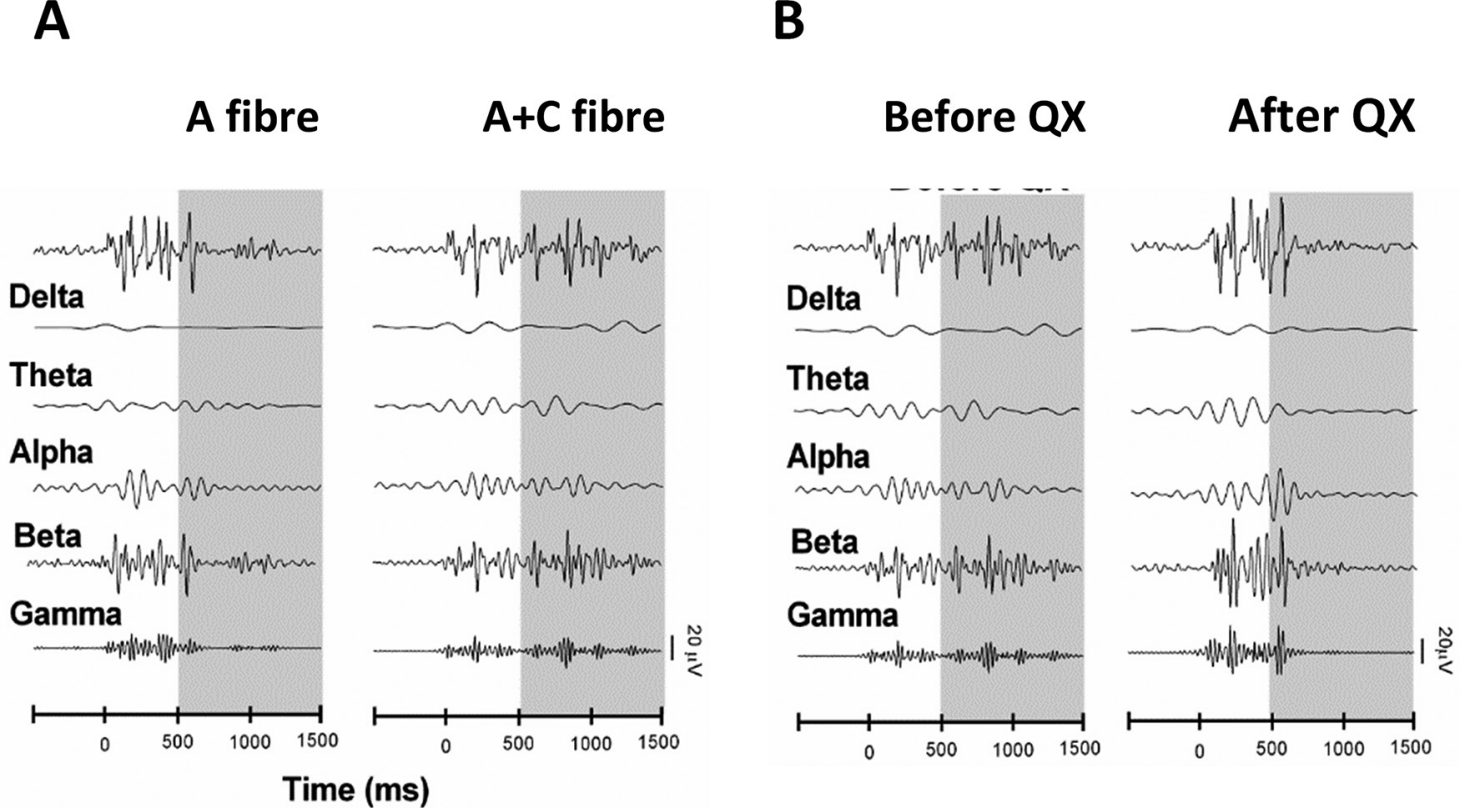
Sample raw traces of single EPs in P30 rat S1, following stimulation of the contralateral hindpaw, filtered to display the contribution from five frequency bands. ***A***, Stimulation at A fiber strength (left) and A + C fiber strength (right). ***B***, Stimulation before (left) and after QX-314 treatment (right).

The main contribution to the energy difference at P30 500–1000 ms following stimulation was a significant increase in the γ band (paired mean diff. 3.0 [95.0%Confidence interval (CI) 0.78, 6.4], two-sided permutation *t* test, *p* < 0.001). This increase in γ energy was also accompanied by increases in α (paired mean diff. 2.4 [95.0%CI 0.83, 2.67], *p* = 0.03) and β (paired mean diff. 1.7 [95.0%CI 1.78, 5.68], *p* < 0.001) band energies (Fig. 3). Figure 4*A* is a sample trace of the oscillatory signals recorded from S1 in a P30 rat following A fiber and at A + C fiber stimulation and shows the increase in γ, α, and β frequency energies caused by recruitment of C fibers. At younger ages, significant decreases in energy were associated with C fiber stimulation: at P7 in the α band at 500–1000 ms (paired mean diff. −2.2, [95.0%CI −4.01, 0.78], *p* = 0.06), and at P21 in the β band energy at 1000–1500 (paired mean diff. −0.67, [95.0%CI −1.23, 0.19], *p* = 0.03; Fig. 3). Those changes were not sufficient to cause a significant drop in overall energy (Fig. 2).

### Silencing C fibers confirms age-related C fiber driven γ and β oscillatory activity in S1

**Figure 5.**
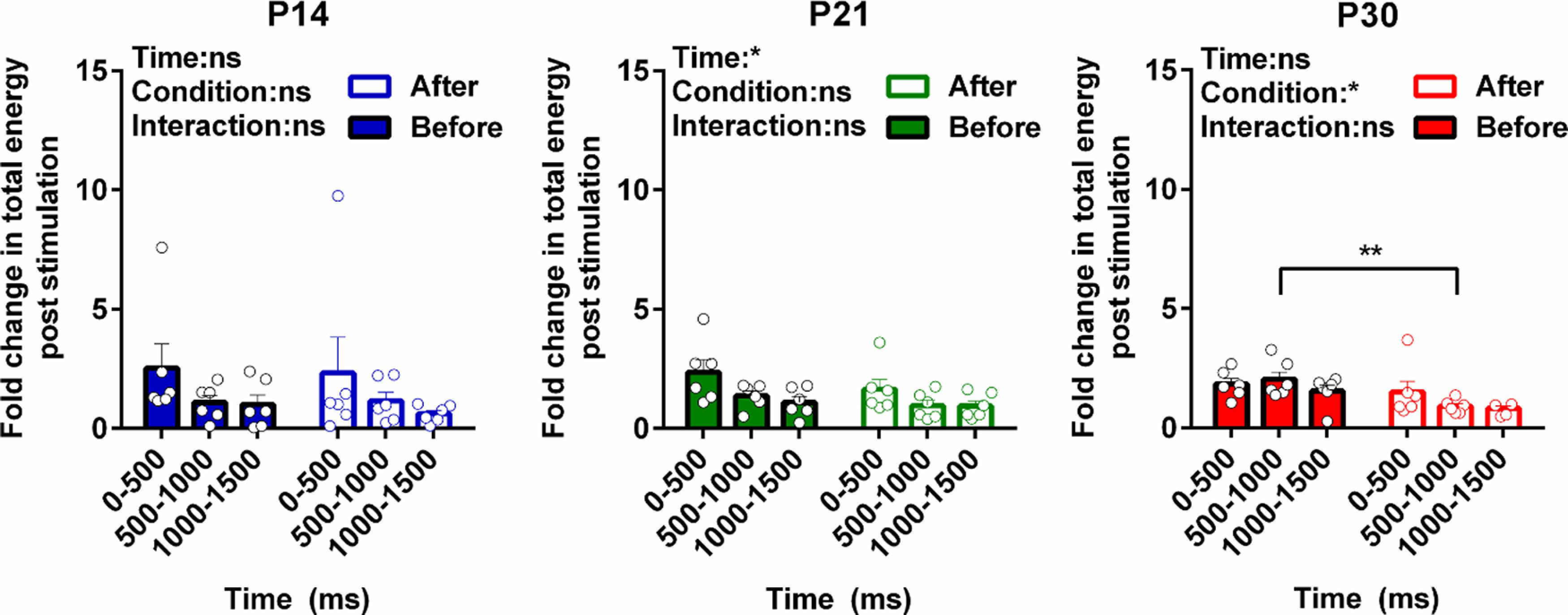
Developmental regulation of total energy changes in S1 neuronal oscillations following C fiber silencing. Total energy changes evoked by A + C fiber (500 μs) electrical stimulation of the contralateral hindpaw at P14, P21, and P30 between 0 and 500, 500 and 1000, and 1000 and 1500 ms poststimulation before and after silencing C fibers input. Mean (±SEM) total energy changes (compared with baseline) to electrical stimuli (10 stimuli at 10-s ISI, 3.2 mA, *n* = 6 rats at each age). Each dot represents the average across stimuli for each rat. Statistical analysis was performed using two-way RM ANOVA (summary result is shown on top left of each panel) with Sidak’s multiple comparisons test (significant between groups were indicated by brackets); *p < 0.05, **p < 0.01, and ns: not significant.

The results above suggest that a distinct pattern of synchronized neural oscillations in the S1 can be attributed to the recruitment of peripheral C fibers. To explore this further, and better understand the age-related changes, we selectively silenced afferent TRPV1 positive C fibers in the hindpaw at P14, P21, and P30 using the membrane impermeable sodium channel blocker QX-314 inserted into C fiber afferents via the TRPV1 channel pore (Binshtok et al., 2007; Puopolo et al., 2013). Effective silencing was confirmed in awake and anaesthetized rats at all ages by significant reduction in hindpaw noxious heat sensitivity and in noxious mechanical reflexes (see Materials and Methods). We first measured the effect of C fiber silencing on the total oscillatory energy in the 1500 ms following stimulation, by comparing the energy in each frequency band evoked before and after QX-314 administration at P14, P21, and P30. Figure 5 shows that the overall energy is significantly reduced by C fiber silencing at P30, but not at younger ages [two-way repeated measures (RM) ANOVA: P30, *p*_condition_ = 0.02, *F*_(1,5)_ = 10.36; P21, *p*_condition_ = 0.12, *F*_(1,5)_ = 3.426; P14, *p*_condition_ = 0.51, *F*_(1,5)_ = 0.49], although peripheral C fibers had been effectively blocked at all ages (see Materials and Methods). C fiber silencing has no significant effect at P14 and P21.

We next tested which frequency bands were contributing to the total energy differences after C fibers silencing in Figure 5. To do this, we compared the energy changes following A + C stimulation after QX-314 treatment with those before treatment, within the same animal, in each frequency band. Data were expressed as the paired difference between energy after and energy before QX-314 at 0–500, 500–1000, and 1000–1500 ms poststimulus, at each age. The data were analyzed using estimation statistics with a permutation *t* test.

**Figure 6.**
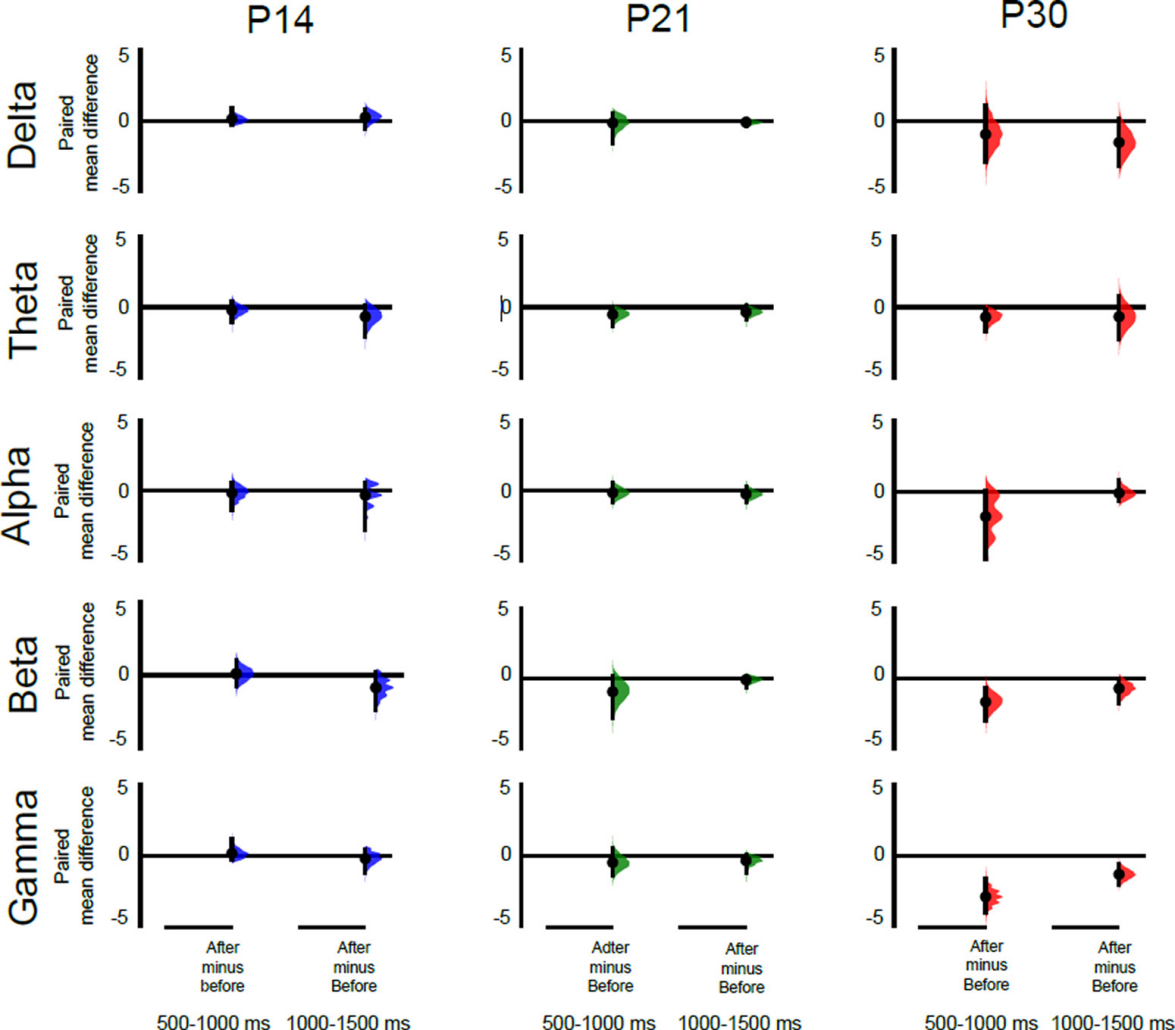
Silencing cutaneous C fibers causes distinct age-related decreases in S1 γ and β oscillatory energy. Differences in the energy evoked by stimulation after QX-314 treatment versus before treatment in P14, P21, and P30 S1 (*n* = 6 animals per age). The paired mean difference at 500–1000 and 1000–1500 ms poststimulus are shown for each frequency band [δ (2–4 Hz), θ (5–7 Hz), α (8–12 Hz), β (15–29 Hz), and γ (30–90 Hz)]. Mean differences are depicted as dots; 95% confidence intervals are indicated by the ends of the vertical error bars. A negative value indicates a decreased energy following QX treatment compared before treatment and can be attributed to silencing of C fibers. Significant decreases in γ and β energy occur at P30. A decrease in β energy at P21 at 0–500 ms is not shown. See text for effect sizes, confidence intervals and permutation *p* values. Each paired mean difference is plotted as a bootstrap sampling distribution, using 5000 bootstrap samples, and the confidence intervals were bias corrected and accelerated (Ho et al., 2019).

Significant decreases in energy occurred at P30, in both the 500- to 1000- and the 1000- to 1500-ms epochs, with the greatest decrease being in the γ band (500–1000 ms, paired mean diff. −2.86 [95.0%CI −3.96, −1.64], two-sided permutation *t* test, *p* < 0.001; 1000–1500 ms, paired mean diff. −1.3 [95.0%CI −2.00, −0.62], two-sided permutation *t* test, *p* = 0.03). This is accompanied by a significant decrease in β band energy (paired mean diff. −1.6 [95.0%CI −2.9, −0.66], *p* = 0.06; Fig. 6). Figure 4*B* is a sample trace of the oscillatory signals recorded from S1 in a P30 rat before and after QX-314 treatment and shows the decrease in γ and β frequency energies caused by silencing C fibers.

At younger ages, no change in γ energy was detected but at P21 a significant decrease in β energy occurred in the first 500 ms (paired mean diff. −1.2, [95.0%CI −2.76, −0.4], *p* < 0.001). No significant changes were detected following C fiber silencing at P14.

## Discussion

Here, we show that in the young adult rat, activation of peripheral cutaneous afferent A and C fibers evokes a distinct age-related oscillatory activity in S1 that differs from that evoked by activation of cutaneous afferent A fibers alone. This was demonstrated by two experiments, one which tested the effect of recruiting C fibers to the stimulation paradigm and one which tested the effect of silencing C fibers in the stimulation paradigm. The effect of recruiting C fibers was shown by the difference between S1 oscillatory activity evoked by peripheral A fibers only and that evoked by A + C fiber stimulation. This difference revealed long latency increases in power in the α, β, and γ range at P30, which can be ascribed to the additional recruitment of C fibers over A fibers alone. The effect of C fiber silencing was shown by the difference between S1 oscillatory activity after and before QX-314/capsaicin treatment. This difference resulted in the loss of long latency γ and β energy, which can be ascribed to the absence of C fiber-driven activity. α Activity was highly variable in the silencing experiment, and no significant change was observed.

The results also show that the segregation of A fiber and C fiber driven oscillatory activity in S1 emerges postnatally. Distinct C fiber-driven γ and β oscillatory activity was only observed between three and four weeks of age (P21–P30); no distinct C fiber oscillatory activity was detected in S1 at younger ages. This finding is consistent with the results from ECoG recording S1 in the awake, freely moving rat pup, where no prolonged change in oscillatory energy occurred after skin incision until P21 (Chang et al., 2016). At P7, the youngest age investigated here, cutaneous C fibers central terminals have grown into the dorsal horn but the maturation of functional synapses with dorsal horn neurons is still taking place (Fitzgerald, 2005), shifting from predominantly Aβ and Aδ fibers innervation to include C fibers innervation (Fitzgerald and Jennings, 1999; Park et al., 1999; Koch and Fitzgerald, 2013). In the following week, from P8 to P14, C fibers evoke increasing spike activity in dorsal horn neurons (Jennings and Fitzgerald, 1998) and brainstem nuclei (Schwaller et al., 2016). The onset of C fibers mediated oscillatory activity in S1 might be expected to occur soon after and the results here show that it is not detectable before P21–P30. The lack of distinct C fiber-driven activity cannot be simply ascribed to the immaturity of neurons in the rat S1 cortex as clear tactile sensory EPs and somatosensory maps can be detected from the end of the first postnatal week (Khazipov et al., 2004; Minlebaev et al., 2011; Mitrukhina et al., 2015). The delayed appearance of distinct C fiber-evoked encoding within the S1 oscillatory activity may reflect the structural and functional maturation of activity-dependent cortical connections over this critical time period (Feldman and Brecht, 2005; Pinto et al., 2013; Pan-Vazquez et al., 2020), in addition to changes in the dorsal horn and thalamus (Koch and Fitzgerald, 2013; Murata and Colonnese, 2019).

In these experiments, we used local field potential recording to compare the effect of electrical stimulation of cutaneous A and C fibers on oscillatory activity in layer 5 of the hindpaw S1. Selective activation of A and A + C fibers with electrical stimulation of different pulse width or current intensity is a classical method in electrophysiology which is still commonly used (Fitzgerald, 1985; Stanfa and Dickenson, 2004; Smith et al., 2020). Higher current intensities are required to excite unmyelinated fibers than myelinated fibers, but because of the strength–duration characteristic of nerve excitation, increasing the duration of a stimulus while keeping amplitude constant allowed us to activate the C fiber component of a CAP, as confirmed by our previous studies (Koch and Fitzgerald, 2014). The technique also allowed us to quantitatively distinguish between A and C fiber evoked activity at different ages using QX-314 C fiber silencing (Binshtok et al., 2007; Koch et al., 2012; Koch and Fitzgerald, 2014). Many natural physiological stimuli will activate both afferent fiber populations, but here we observed the difference between the composite effect of activating A and C fibers simultaneously versus A fibers alone, so it is the “additive” contribution of the C fibers in a scenario where both A and C fibers have been engaged. Silencing the C fibers revealed the effect of removing that additive contribution. Selective activation of C fibers alone using optogenetics (Beaudry et al., 2017) would discount any interaction caused by simultaneous stimulation of A and C fibers, if the same time locked frequency plots could be achieved at all ages.

The pattern of C fiber evoked γ activity reported here is consistent with reports of γ oscillatory activity associated with noxious laser stimulation in humans and rats. Its latency of >500 ms poststimulus is consistent with the slow conduction velocity of C fibers. Pain-related γ oscillatory activity in the somatosensory S1 predicts pain sensitivity (Hauck et al., 2007; Hu and Iannetti, 2019; Heid et al., 2020) and inducing γ oscillations in S1 enhances nociceptive sensitivity and induces aversive avoidance behavior (Tan et al., 2019). Furthermore, noxious laser evoked spiking in superficial S1 interneurons has strong phase coherence with γ oscillations in awake rats (Yue et al., 2020). C fiber activity was also associated with increases in β energy at >500-ms latency poststimulus. In human subjects noxious stimulus intensity is encoded by decreases of neuronal oscillations at α and β frequencies in sensorimotor areas (Nickel et al., 2017) but the β (20–30 Hz) oscillations observed in our study may be related to the gating of C fiber input. Recent optogenetic studies show that β oscillations are generated in S1 by strong feedback from the secondary somatosensory thalamus, which contains subpopulations of neurons that are highly responsive to noxious stimuli (Zhang and Bruno, 2019). An *in vivo* study of anesthetized mice found that this thalamic input can enhance the responsiveness of L5 pyramidal neurons to sensory stimulation (Mease et al., 2016). Thus the appearance of C fiber-driven β oscillations might increase the salience of somatic sensory inputs, especially when combined with γ oscillations (Whittington et al., 2018). Recruiting C fibers in our study also increased α band energy consistent with reports in mice with inflammatory pain showing elevated resting α as well as γ activity (Tan et al., 2019), but α activity was not sensitive to C fiber silencing and is therefore not likely to be directly linked to C fiber inputs.

However, it is important to note that the oscillatory profile reported here is selective for C fiber afferent inputs but not necessarily selective for pain. The electrical stimulation paradigm separates the two major afferent fiber groups, myelinated A fibers and unmyelinated C fibers, but does not separate nociceptive and non-nociceptive modalities. While the majority of rodent sensory C fibers are polymodal nociceptors, the fundamental driver of lasting, unpleasant pain (Chisholm et al., 2018), some plantar foot C fibers in rats are cold receptors (but not low threshold mechanoreceptors as in other skin regions); equally, many nociceptors are Aδ fibers (Leem et al., 1993).

These experiments were performed under light inhalation anesthesia as stimulation at intensities required to activate C fibers evokes strong reflex movements in awake animals that would confound the results and cause considerable stress. Importantly, peripheral afferent volleys, traveling through ascending tracts, are known to reach the S1 under anesthesia in man (where they are used to improve accuracy in neurosurgery; Eseonu et al., 2017) and in adult and infant rats (Schouenborg et al., 1986; Kalliomäki et al., 1993, 1998; Shaw et al., 2001; Granmo et al., 2013; Chang et al, 2015, 2016). In rat pups, isoflurane at 1.5% does not affect the amplitude or frequency content of the initial somatosensory potential evoked by whisker deflection, but suppresses the successive early γ oscillations (EGO) and spindle bursts (Minlebaev et al., 2011; Sitdikova et al., 2014), which are the result of corticothalamic feedback loop synchronization. This suggests that isoflurane does not interfere with the arrival of the afferent volley to S1 but inhibits further cortical processing. This effect may also differ with age (Chang et al., 2015); indeed, somatosensory and noxious-evoked cortical activity in young animals is highly resistant to anesthesia (Sitdikova et al., 2014; Chang et al., 2016), which may explain the very high oscillatory S1 energies, relative to baseline, recorded here at P7. Our data are presented as differences within the same aged animals under the same level of anesthesia, rather than between ages, because of this possibility. While the implications of these data for cortical mechanisms of sensory perception are limited by the anesthesia, this does not diminish the importance of the information on encoding of afferent volleys in S1.

This study may have implications for the development of human cortical somatosensory processing. Behavioral reflex recording in newborn infants reveal that nociceptive reflexes are indistinguishable from those evoked by innocuous touch (Fitzgerald et al., 1988; Cornelissen et al., 2013). The human infant brain undergoes a transition in response to tactile and noxious stimulation from nonspecific, evenly dispersed neuronal bursts to modality-specific, localized, EPs, suggesting that specific neural circuits necessary for discrimination between touch and nociception emerge from 35 to 37 weeks gestation (Fabrizi et al., 2011). The emergence of the behavioral discrimination in early human life coincides with the brain responses discriminating noxious and innocuous events (Green et al., 2019), suggesting a potential mechanistic link. By term, distinct BOLD activity patterns can be measured in response to different modalities and intensities of skin sensory stimulation in infants (Williams et al., 2015), but comparison of EEG responses to the same time-locked noxious skin lance revealed distinct differences in adult and infant oscillatory activity still remain (Fabrizi et al., 2016). This is consistent with the results of the current study where a direct and systematic measure of selective C fiber afferent encoding in rat S1 cortex is shown to change with age.

In conclusion, the results show that peripheral C fiber activity modulates oscillatory energy in the young adult rat S1, producing a distinct signature of increased β and γ rhythms, not observed following A fiber stimulation alone. Furthermore, it demonstrates the prolonged postnatal maturation of C fiber afferent coding in the mammalian brain.

## Synthesis

Reviewing Editor: Karen Davis, Krembil Research Institute and University of Toronto Decisions are customarily a result of the Reviewing Editor and the peer reviewers coming together and discussing their recommendations until a consensus is reached. When revisions are invited, a fact-based synthesis statement explaining their decision and outlining what is needed to prepare a revision will be listed below. The following reviewer(s) agreed to reveal their identity: NONE.

All concerns were adequately addressed.

## Author Response

Reply to Referees eNeuro eN-NWR-0036-20R1

We thank the referees for their new list of corrections, all of which have been addressed below.

Furthermore, we recognise that the emphasis on pain processing was not appropriate and that the message of this paper is really about the encoding of A and C fibre afferent volleys in S1. We also recognise that we did not discuss the role of anaesthesia sufficiently or consider other technical limitations. As a result, we have substantially revised the Abstract, Introduction and Discussion to reflect these shortcomings (marked red in the revised ms).

Please note that Referee point 3 (major concern) is incorrect. Our null hypothesis is not that changes at P30 and P14 are the same. Our null hypothesis is that there is no change in gamma at any given age. Our test is appropriate for this second more crucial hypothesis. Indeed, rejecting the former null hypothesis would be inconclusive.

Synthesis Statement for Author (Required):

The authors appropriately addressed some of the previous concerns and added new experiments and results. However, major concerns remain.

1. A major issue related to anesthesia and stimulation paradigm was not answered properly: The relevance of C fibers to pain is an obvious one, but there is no pain under anesthesia. There’s no behavioral evidence of pain, only suggestive electrophysiological data. To counter this critique, the authors emphasize that this is essentially a study of C-fiber evoked connectivity in S1, i.e. not pain processing per se. “We have added to this in the Discussion and the importance of within, rather than between, age comparisons” , however the assumptions about cortical ‘pain’ processing percolate throughout the manuscript (the word pain is mentioned 38x). A reader will eventually walk away with the wrong message that this paper is indeed about pain processing in S1. More importantly, the question of whether the effects reported are due to anesthetics acting differentially on young vs. older rats was not addressed, and the answer provided “added in the Discussion the importance of within, rather than between, age comparisons” is not satisfactory. In fact, Fig 1, 6 and 7 compare between ages.

We agree that our results do not show that the energy changes observed are related to pain perception, but rather with the difference in encoding the incoming A and A+C volleys. We have now removed the focus on pain from the manuscript and only mention possible applications to cortical pain processing at the end of the Discussion.

Afferent volleys, travelling through ascending tracts, are known to reach the primary somatosensory cortex under anaesthesia in man (where they are used to improve accuracy in neurosurgery (for example, Eseonu CI, et al., Neurosurgery 2017) and in adults and infant rats (Granmo et al., 2013; Kalliomäki et al., 1998, 1993; Schouenborg et al., 1986; Shaw et al., 2001; Chang et al., 2016). In rat pups isoflurane at 1.5% does not affect the amplitude or frequency content of the initial somatosensory potential evoked by whisker deflection, but suppresses the successive early gamma oscillations (EGO) and spindle bursts (Minlebaev et al., 2011; Sitdikova et al., 2014) which are the result of corticothalamic feedback loop synchronization. This suggests that isoflurane does not interfere with the arrival of the afferent volley to SI but inhibits further cortical processing. In summary, the propagation of this information to higher cortical centres is, we agree, less clear under anaesthesia. To clarify this point, we now emphasise in the Abstract, Introduction and Discussion that the ms. reports the difference in encoding of A fibre and C fibre afferent volleys by the subsequent SI oscillatory rhythms and is not a study of cortical pain networks.

Regarding differences across ages. Our null hypothesis is not that energy changes following A or A+C fibre stimulation at P30, P21, P14 and P7 are the same. Our null hypothesis is that there is no change in any frequency band at any given age. Our test is appropriate for this actual, more crucial hypothesis. Indeed rejecting the former null hypothesis would be inconclusive. We have removed the only age comparison from the paper (Figure 1). Figure 6 and 7 did not have age comparisons.

2. Another issue related to anesthesia and stimulation paradigm that was also not answered properly is related to pain vs. no-pain. A previous question asked “It is not clear, whether these signals reflect pain processing per se as innocuous stimuli do not appear to have been tested”. The authors response to this question is not accurate “The design of the experiment was to directly compare A fibre stimulation, which is innocuous because only low intensities that recruit low threshold afferent fibre groups were used, with C fibre stimulation, which is noxious because it recruits high threshold afferent fibre groups”. A fibers contain A-delta, some of which relay ‘fast pain’, and not all C fibers are ‘painful’. Moreover, justification for the electrical stimulation paradigm (pulse width 50 vs. 500 mics at the same amplitude of 3.2 mA guarantees selectivity) is referred to an older citation (Koch 2014), which is based on single unit recordings from the dorsal horn, not the more conclusive CAP method. Effects of wind-up were not tested. This leads to another previous question about selectivity of afferent stimulation, and the recommendation to use readily available optogenetic tagging methods. Instead, the authors added new experiments using mechanical brush and pinch. Unfortunately, this change is in the wrong direction due to inherently poor selectivity and temporal resolution of these stimuli. This also ties in with the caveat of making assumptions about C fibers EP by ‘subtraction’ from the A+C fibers because there is no guarantee that the incoming A fiber volley is not biasing the brain state by the time the slower C fiber volley hits. The only way to do this properly is to selectively drive C fibers, and at a high temporal precision, a technically feasible experiment.

These are important points and have been addressed below and where appropriate have been added to the revised the ms.

Selective activation of A and A+C fibres with electrical stimulation of different pulse width or current intensity is a classical method in electrophysiology which is still used today (Fitzgerald, J Physiol 1985, Stanfa & Dickenson Methods Mol Med 2004, Smith et al., J Neurosci Methods 2020). Higher current intensities are required to excite unmyelinated fibres than myelinated fibres, but because of the Strength-Duration characteristic of nerve excitation, increasing the duration of a stimulus while keeping amplitude constant activates the C fibre component of a compound action potential (CAP) (see Brocker & Grill Brain Stimulation, Handbook of Clinical Neurology, 2013 for further explanation). CAP were indeed measured in Koch 2014 (not only single unit recordings) to confirm the parameters used here for selective activation of fast and slow conducting fibres. We have now added extra explanatory references to our manuscript.

Wind up, the well-known phenomenon where spinal cord neurons fire increasingly to the same strength stimulus only occurs if there is a short inter-stimulus gap of less than 2-3 secs (Mendell, 1966, Smith et al, 2020), while in this study stimuli were applied at 10 sec intervals.

Since this paper is only about differential encoding of afferent fibre input (which can be precisely controlled with electrical stimulation), we have removed the mechanical stimulation results and will write the data up using natural stimulation separately.

We agree that the initial arrival of the A fibre volley could somehow bias the brain before the arrival of the C fibre volley, indeed this is the case at the level of the spinal cord and was the basis of the gate theory (Melzack & Wall, 1965). It is also true that many physiological stimuli will activate both afferent fibre populations. What we observed here is the difference between the composite effect of activating A and C fibre simultaneously vs A fibres alone, so it is the “additive” contribution of the C fibres in a scenario where both A and C fibres have been engaged. Silencing the C fibres shows the removal of that additive contribution. It is true that this is not the same as pure activation of C fibres alone and we have now made this clear in the Discussion.

As explained above, activation of peripheral afferent fibres with electrical stimulation is a classic, well controlled, and accepted method which has been used in the field for many years. We completely agree that optogenetic tagging and stimulation would have the advantage of providing ‘pure’ C fibre afferent volleys, but this technique would not have the temporal resolution required for analysing stimulus evoked brain oscillations in this way. In addition, the technique would need to be developed for all the postnatal ages tested here. We hope that the reviewer and Reviewing Editor will agree that we cannot add these experiments at this time.

3. Another major remaining concern is that the study does not really provide convincing statistical evidence regarding how c-fibre evoked oscillations develop with age (stated goals on lines 67-69). That is not to say the data is not presented, but instead that the relevant analyses have yet to be performed. For example, while it certainly appears that Alpha oscillations are tied to c fibre activation on Day 30 but not Day 14 - no explicit statistical test of this fact is performed (i.e. comparison of alpha changes on Days 30 and 14). This did not occur to me in the first read, but now that the paper has been cleaned up significantly it appears more starkly. As I have indicated to the editor, I am torn as to whether fair conclusions can be drawn from the figures or whether additional analyses are absolutely necessary.

As mentioned above our null hypothesis is that there is no change in energy for any frequency band at any age. The example mentioned by the reviewer is exactly tested with the current version of statistical test. If we were to compare alpha changes across ages and find a significant difference, the statement would read as “changes in alpha band energy are larger at P30 than at P14” however this test would not tell us if changes in alpha energy are significant at either ages, which is what we are interested in. Also, as requested by the reviewer, direct comparison of amplitudes between ages was not the aim here.

4. Some minor additional minor comments:

- It is unclear what corrections for multiple comparisons have been applied to the TFRs presented in Figure 7. The paper methods suggest that details can be found in another manuscript, but I believe those details should be provided in the current manuscript as well.

This data has been removed from the ms., as we are now focussing only on afferent volleys in response to the Referee’s concerns.

- The significant main effect of time on P21 in the C-fibre inactivation experiments appears to be misinterpreted (lines 294-295). The main effect, in the absence of a time x condition interactions, suggests that the effect time is equal in both conditions (block vs no block) and therefore cannot suggest that, “silencing alters the post-stimulus timing”. I would also suggest adjusting Figure 5 legends to make outliers present on P14 more easily seen.

This is correct and thank you for pointing this out. The test shows that C fibres silencing has no effect at P14. The data suggests a trend towards A+C fibre stimulation evoking a larger energy change 0-500 ms post-stimulus compared to later time points, but there is no significant interaction between time x stimulus.

We have edited Figure 5 so that the outliers are more clearly seen.

- A few typographical errors are present in the manuscript:

a. line 57 contains an extraneous “and"

b. Line 220 appears to have an incorrect p value (p <.5) and appears incompletely written. These have been corrected.

c. In figure 1 legend: are C & D really analyzed with a 2-way ANOVA? It appears that they could only be a one way ANOVA (main effect of time), unless the factor of modality (Brush vs. Pinch) is being included. I could not find this in the manuscript, so I suspect this is an error.

This figure has been removed from the ms. as we are now focussing on afferent volleys only.

## References

[B1] Baccei ML, Fitzgerald M (2004) Development of GABAergic and glycinergic transmission in the neonatal rat dorsal horn. J Neurosci 24:4749–4757. 10.1523/JNEUROSCI.5211-03.2004 15152035 PMC6729459

[B2] Baccei ML, Fitzgerald M (2013) Development of pain pathways and mechanisms In: Wall and Melzack’s textbook of pain (McMahon SB, Koltzenburg M, Tracey I, Turk D, eds), pp 143–155. New York: Elsevier.

[B3] Baccei ML, Fitzgerald M, Fitzgerald M (2003) Development of nociceptive synaptic inputs to the neonatal rat dorsal horn: glutamate release by capsaicin and menthol. J Physiol 549:231–242. 10.1113/jphysiol.2003.040451 12679376 PMC2342935

[B4] Beaudry H, Daou I, Ase AR, Ribeiro-da-Silva A, Séguéla P (2017) Distinct behavioral responses evoked by selective optogenetic stimulation of the major TRPV1+ and MrgD+ subsets of C-fibers. Pain 158:2329–2339. 10.1097/j.pain.0000000000001016 28708765

[B5] Beggs S, Torsney C, Drew LJ, Fitzgerald M (2002) The postnatal reorganization of primary afferent input and dorsal horn cell receptive fields in the rat spinal cord is an activity-dependent process. Eur J Neurosci 16:1249–1258. 10.1046/j.1460-9568.2002.02185.x 12405985

[B6] Binshtok AM, Bean BP, Woolf CJ (2007) Inhibition of nociceptors by TRPV1-mediated entry of impermeant sodium channel blockers. Nature 449:607–610. 10.1038/nature06191 17914397

[B7] Bruns A (2004) Fourier-, Hilbert- and wavelet-based signal analysis: are they really different approaches? J Neurosci Methods 137:321–332. 10.1016/j.jneumeth.2004.03.002 15262077

[B8] Chang P, Walker SM, Fitzgerald M (2015) Neonatal rat primary somatosensory cortical pain activity is resistant to isoflurane anesthesia. Anesthesiology 124:885–898. 10.1097/ALN.0000000000001017 26808637

[B9] Chang P, Fabrizi L, Olhede S, Fitzgerald M (2016) The development of nociceptive network activity in the somatosensory cortex of freely moving rat pups. Cereb Cortex 26:4513–4523. 10.1093/cercor/bhw330 27797835 PMC5193146

[B10] Chisholm KI, Khovanov N, Lopes DM, La Russa F, McMahon SB (2018) Large scale in vivo recording of sensory neuron activity with GCaMP6. eNeuro 5:ENEURO.0417-17.2018 10.1523/ENEURO.0417-17.2018 PMC5898788 29662940

[B11] Coggeshall RE, Jennings EA, Fitzgerald M (1996) Evidence that large myelinated primary afferent fibers make synaptic contacts in lamina II of neonatal rats. Brain Res Dev Brain Res 92:81–90. 10.1016/0165-3806(95)00207-3 8861726

[B12] Cornelissen L, Fabrizi L, Patten D, Worley A, Meek J, Boyd S, Slater R, Fitzgerald M (2013) Postnatal temporal, spatial and modality tuning of nociceptive cutaneous flexion reflexes in human infants. PLoS One 8:e76470 10.1371/journal.pone.0076470 24124564 PMC3790695

[B13] Eseonu CI, Rincon-Torroella J, ReFaey K, Lee YM, Nangiana J, Vivas-Buitrago T, Quiñones-Hinojosa A (2017) Awake craniotomy vs craniotomy under general anesthesia for perirolandic gliomas: evaluating perioperative complications and extent of resection. Neurosurgery 81:481–489. 10.1093/neuros/nyx023 28327900

[B14] Fabrizi L, Slater R, Worley A, Meek J, Boyd S, Olhede S, Fitzgerald M (2011) A shift in sensory processing that enables the developing human brain to discriminate touch from pain. Curr Biol 21:1552–1558. 10.1016/j.cub.2011.08.010 21906948 PMC3191265

[B15] Fabrizi L, Verriotis M, Williams G, Lee A, Meek J, Olhede S, Fitzgerald M (2016) Encoding of mechanical nociception differs in the adult and infant brain. Sci Rep 6:28642. 10.1038/srep28642 27345331 PMC4921818

[B16] Feldman DE, Brecht M (2005) Map plasticity in somatosensory cortex. Science 310:810–815. 10.1126/science.1115807 16272113

[B17] Fitzgerald M (1985) The post-natal development of cutaneous afferent fibre input and receptive field organization in the rat dorsal horn. J Physiol 364:1–18. 10.1113/jphysiol.1985.sp015725 4032293 PMC1192950

[B18] Fitzgerald M (2005) The development of nociceptive circuits. Nat Rev Neurosci 6:507–520. 10.1038/nrn1701 15995722

[B19] Fitzgerald M, Jennings E (1999) The postnatal development of spinal sensory processing. Proc Natl Acad Sci USA 96:7719–7722. 10.1073/pnas.96.14.7719 10393887 PMC33608

[B20] Fitzgerald M, Shaw A, MacIntosh N (1988) Postnatal development of the cutaneous flexor reflex: comparative study of preterm infants and newborn rat pups. Dev Med Child Neurol 30:520–526. 10.1111/j.1469-8749.1988.tb04779.x 3169392

[B21] Fransen AMM, Dimitriadis G, van Ede F, Maris E (2016) Distinct α- and β-band rhythms over rat somatosensory cortex with similar properties as in humans. J Neurophysiol 115:3030–3044. 10.1152/jn.00507.2015 27009160 PMC4922619

[B22] Granmo M, Petersson P, Schouenborg J (2008) Action-based body maps in the spinal cord emerge from a transitory floating organization. J Neurosci 28:5494–5503. 10.1523/JNEUROSCI.0651-08.2008 18495883 PMC6670632

[B23] Granmo M, Jensen T, Schouenborg J (2013) Nociceptive transmission to rat primary somatosensory cortex–comparison of sedative and analgesic effects. PLoS One 8:e53966. 10.1371/journal.pone.0053966 23320109 PMC3540052

[B24] Green G, Hartley C, Hoskin A, Duff E, Shriver A, Wilkinson D, Adams E, Rogers R, Moultrie F, Slater R (2019) Behavioural discrimination of noxious stimuli in infants is dependent on brain maturation. Pain 160:493–500. 10.1097/j.pain.0000000000001425 30422872 PMC6343955

[B25] Hauck M, Lorenz J, Engel AK (2007) Attention to painful stimulation enhances γ-band activity and synchronization in human sensorimotor cortex. J Neurosci 27:9270–9277. 10.1523/JNEUROSCI.2283-07.2007 17728441 PMC6673131

[B26] Heid C, Mouraux A, Treede R-D, Schuh-Hofer S, Rupp A, Baumgärtner U (2020) Early gamma-oscillations as correlate of localized nociceptive processing in primary sensorimotor cortex. J Neurophysiol 123:1711–1726. 32208893 10.1152/jn.00444.2019

[B27] Ho J, Tumkaya T, Aryal S, Choi H, Claridge-Chang A (2019) Moving beyond P values: data analysis with estimation graphics. Nat Methods 16:565–566. 10.1038/s41592-019-0470-3 31217592

[B28] Hu L, Iannetti GD (2019) Neural indicators of perceptual variability of pain across species. Proc Natl Acad Sci USA 116:1782–1791. 10.1073/pnas.1812499116 30642968 PMC6358671

[B29] Jennings E, Fitzgerald M (1998) Postnatal changes in responses of rat dorsal horn cells to afferent stimulation: a fibre-induced sensitization. J Physiol 509:859–868. 10.1111/j.1469-7793.1998.859bm.x 9596805 PMC2230995

[B30] Kalliomäki J, Weng HR, Nilsson HJ, Schouenborg J (1993) Nociceptive C fibre input to the primary somatosensory cortex (SI). A field potential study in the rat. Brain Res 622:262–270. 10.1016/0006-8993(93)90827-a 8242365

[B31] Kalliomäki J, Luo XL, Yu YB, Schouenborg J (1998) Intrathecally applied morphine inhibits nociceptive C fiber input to the primary somatosensory cortex (SI) of the rat. Pain 77:323–329. 10.1016/s0304-3959(98)00115-8 9808358

[B32] Khazipov R, Sirota A, Leinekugel X, Holmes GL, Ben-Ari Y, Buzsáki G (2004) Early motor activity drives spindle bursts in the developing somatosensory cortex. Nature 432:758–761. 10.1038/nature03132 15592414

[B33] Killackey HP (1973) Anatomical evidence for cortical subdivisions based on vertically discrete thalamic projections from the ventral posterior nucleus to cortical barrels in the rat. Brain Res 51:326–331. 10.1016/0006-8993(73)90383-1 4706020

[B34] Koch SC, Fitzgerald M (2013) Activity-dependent development of tactile and nociceptive spinal cord circuits. Ann NY Acad Sci 1279:97–102. 10.1111/nyas.12033 23531007

[B35] Koch SC, Fitzgerald M (2014) The selectivity of rostroventral medulla descending control of spinal sensory inputs shifts postnatally from A fibre to C fibre evoked activity. J Physiol 592:1535–1544. 10.1113/jphysiol.2013.267518 24421353 PMC3979610

[B36] Koch SC, Tochiki KK, Hirschberg S, Fitzgerald M (2012) C-fiber activity-dependent maturation of glycinergic inhibition in the spinal dorsal horn of the postnatal rat. Proc Natl Acad Sci USA 109:12201–12206. 10.1073/pnas.1118960109 22778407 PMC3409769

[B37] Le Van Quyen M, Foucher J, Lachaux J, Rodriguez E, Lutz A, Martinerie J, Varela FJ (2001) Comparison of Hilbert transform and wavelet methods for the analysis of neuronal synchrony. J Neurosci Methods 111:83–98. 10.1016/s0165-0270(01)00372-7 11595276

[B38] Leem JW, Willis WD, Chung JM (1993) Cutaneous sensory receptors in the rat foot. J Neurophysiol 69:1684–1699. 10.1152/jn.1993.69.5.1684 8509832

[B39] Mease RA, Metz M, Groh A (2016) Cortical sensory responses are enhanced by the higher-order thalamus. Cell Rep 14:208–215. 26748702 10.1016/j.celrep.2015.12.026

[B40] Minlebaev M, Colonnese M, Tsintsadze T, Sirota A, Khazipov R (2011) Early gamma oscillations synchronize developing thalamus and cortex. Science 334:226–229. 10.1126/science.1210574 21998388

[B41] Mitrukhina O, Suchkov D, Khazipov R, Minlebaev M (2015) Imprecise whisker map in the neonatal rat barrel cortex. Cereb Cortex 25:3458–3467. 10.1093/cercor/bhu169 25100857

[B42] Murata Y, Colonnese MT (2019) Thalamic inhibitory circuits and network activity development. Brain Res 1706:13–23. 10.1016/j.brainres.2018.10.024 30366019 PMC6363901

[B43] Nickel MM, May ES, Tiemann L, Schmidt P, Postorino M, Ta Dinh S, Gross J, Ploner M (2017) Brain oscillations differentially encode noxious stimulus intensity and pain intensity. Neuroimage 148:141–147. 10.1016/j.neuroimage.2017.01.011 28069543 PMC5349759

[B44] Pan-Vazquez A, Wefelmeyer W, Gonzalez Sabater V, Neves G, Burrone J (2020) Activity-dependent plasticity of axo-axonic synapses at the axon initial segment. Neuron 106:265–276.e6. 10.1016/j.neuron.2020.01.037 32109363 PMC7181187

[B45] Park JS, Nakatsuka T, Nagata K, Higashi H, Yoshimura M (1999) Reorganization of the primary afferent termination in the rat spinal dorsal horn during post-natal development. Brain Res Dev Brain Res 113:29–36. 10.1016/s0165-3806(98)00186-2 10064871

[B46] Paxinos G, Ashwell KWS, Tork I (2013) Atlas of the developing rat nervous system, Ed 3 San Diego, USA: Elsevier.

[B47] Paxinos G, Watson C (2013) The rat brain in stereotaxic coordinates, Ed 7 San Diego, USA: Elsevier.

[B48] Peng W, Tang D (2016) Pain related cortical oscillations: methodological advances and potential applications. Front Comput Neurosci 10. 10.3389/fncom.2016.00009 PMC4740361 26869915

[B49] Peng W, Xia X, Yi M, Huang G, Zhang Z, Iannetti G, Hu L (2018) Brain oscillations reflecting pain-related behavior in freely moving rats. Pain 159:106–118. 10.1097/j.pain.0000000000001069 28953192 PMC5737457

[B50] Pfurtscheller G, Lopes da Silva FH (1999) Event-related EEG/MEG synchronization and desynchronization: basic principles. Clin Neurophysiol 110:1842–1857. 10.1016/s1388-2457(99)00141-8 10576479

[B51] Pinto JGA, Jones DG, Murphy KM (2013) Comparing development of synaptic proteins in rat visual, somatosensory, and frontal cortex. Front Neural Circuits 7:97. 23754984 10.3389/fncir.2013.00097 PMC3664769

[B52] Puopolo M, Binshtok AM, Yao G-L, Oh SB, Woolf CJ, Bean BP (2013) Permeation and block of TRPV1 channels by the cationic lidocaine derivative QX-314. J Neurophysiol 109:1704–1712. 10.1152/jn.00012.2013 23303863 PMC3628012

[B53] Schouenborg J, Kalliomäki J, Gustavsson P, Rosén I (1986) Field potentials evoked in rat primary somatosensory cortex (SI) by impulses in cutaneous A beta- and C-fibres. Brain Res 397:86–92. 10.1016/0006-8993(86)91371-5 3801867

[B54] Schwaller F, Kanellopoulos AH, Fitzgerald M (2017) The developmental emergence of differential brainstem serotonergic control of the sensory spinal cord. Sci Rep 7:2215. 10.1038/s41598-017-02509-2 28533557 PMC5440407

[B55] Schwaller F, Kwok C, Fitzgerald M (2016) Postnatal maturation of the spinal-bulbo-spinal loop: brainstem control of spinal nociception is independent of sensory input in neonatal rats. Pain 157:677–686. 10.1097/j.pain.0000000000000420 26574823 PMC4751743

[B56] Shaw FZ, Chen RF, Yen CT (2001) Dynamic changes of touch- and laser heat-evoked field potentials of primary somatosensory cortex in awake and pentobarbital-anesthetized rats. Brain Res 911:105–115. 10.1016/s0006-8993(01)02686-5 11511377

[B57] Sitdikova G, Zakharov A, Janackova S, Gerasimova E, Lebedeva J, Inacio AR, Zaynutdinova D, Minlebaev M, Holmes GL, Khazipov R (2014) Isoflurane suppresses early cortical activity. Ann Clin Transl Neurol 1:15–26. 10.1002/acn3.16 25356379 PMC4207500

[B58] Smith TM, Lee D, Bradley K, McMahon SB (2020) Methodology for quantifying excitability of identified projection neurons in the dorsal horn of the spinal cord, specifically to study spinal cord stimulation paradigms. J Neurosci Methods 330:108479. 10.1016/j.jneumeth.2019.108479 31705935

[B59] Stančák A (2006) 237–252. Cortical oscillatory changes occurring during somatosensory and thermal stimulation. Prog Brain Res 159:237–252. 17071235 10.1016/S0079-6123(06)59016-8

[B60] Stanfa LC, Dickenson AH (2004) In vivo electrophysiology of dorsal-horn neurons. Methods Mol Med 99:139–153. 10.1007/978-1-59259-770-3_12 15131335

[B61] Tadel F, Baillet S, Mosher JC, Pantazis D, Leahy RM (2011) Brainstorm: a user-friendly application for MEG/EEG analysis. Comput Intell Neurosci 2011:879716. 10.1155/2011/879716 21584256 PMC3090754

[B62] Tan LL, Oswald MJ, Heinl C, Retana Romero OA, Kaushalya SK, Monyer H, Kuner R (2019) Gamma oscillations in somatosensory cortex recruit prefrontal and descending serotonergic pathways in aversion and nociception. Nat Commun 10:983. 10.1038/s41467-019-08873-z 30816113 PMC6395755

[B63] Walker SM, Meredith-Middleton J, Lickiss T, Moss A, Fitzgerald M (2007) Primary and secondary hyperalgesia can be differentiated by postnatal age and ERK activation in the spinal dorsal horn of the rat pup. Pain 128:157–168. 17056180 10.1016/j.pain.2006.09.015

[B64] Vierck CJ, Whitsel BL, Favorov OV, Brown AW, Tommerdahl M (2013) Role of primary somatosensory cortex in the coding of pain. Pain 154:334–344. 23245864 10.1016/j.pain.2012.10.021 PMC4501501

[B65] Welker C (1971) Microelectrode delineation of fine grain somatotopic organization of (SmI) cerebral neocortex in albino rat. Brain Res 26:259–275. 4100672

[B66] Whittington MA, Traub RD, Adams NE (2018) A future for neuronal oscillation research. Brain Neurosci Adv 2:2398212818794827. 10.1177/2398212818794827 32166146 PMC7058255

[B67] Williams G, Fabrizi L, Meek J, Jackson D, Tracey I, Robertson N, Slater R, Fitzgerald M (2015) Functional magnetic resonance imaging can be used to explore tactile and nociceptive processing in the infant brain. Acta Paediatr 104:158–166. 10.1111/apa.12848 25358870 PMC4463763

[B68] Yue L, Iannetti GD, Hu L (2020) The neural origin of nociceptive-induced gamma-band oscillations. J Neurosci 40:3478–3490. 10.1523/JNEUROSCI.0255-20.2020 32241836 PMC7178916

[B69] Zhang W, Bruno RM (2019) High-order thalamic inputs to primary somatosensory cortex are stronger and longer lasting than cortical inputs. Elife 8:e44158 10.7554/eLife.44158 30741160 PMC6370338

